# A novel role of dendritic gap junction and mechanisms underlying its interaction with thalamocortical conductance in fast spiking inhibitory neurons

**DOI:** 10.1186/1471-2202-10-131

**Published:** 2009-10-29

**Authors:** Qian-Quan Sun

**Affiliations:** 1 Department of Zoology and Physiology, University of Wyoming, Laramie, WY 82071, USA

## Abstract

**Background:**

Little is known about the roles of dendritic gap junctions (GJs) of inhibitory interneurons in modulating temporal properties of sensory induced responses in sensory cortices. Electrophysiological dual patch-clamp recording and computational simulation methods were used in combination to examine a novel role of GJs in sensory mediated feed-forward inhibitory responses in barrel cortex layer IV and its underlying mechanisms.

**Results:**

Under physiological conditions, excitatory post-junctional potentials (EPJPs) interact with thalamocortical (TC) inputs within an unprecedented few milliseconds (i.e. over 200 Hz) to enhance the firing probability and synchrony of coupled fast-spiking (FS) cells. Dendritic GJ coupling allows fourfold increase in synchrony and a significant enhancement in spike transmission efficacy in excitatory spiny stellate cells. The model revealed the following novel mechanisms: ***1) ***rapid capacitive current (I_cap_) underlies the activation of voltage-gated sodium channels; ***2) ***there was less than 2 milliseconds in which the I_cap _underlying TC input and EPJP was coupled effectively; ***3) ***cells with dendritic GJs had larger input conductance and smaller membrane response to weaker inputs; ***4) ***synchrony in inhibitory networks by GJ coupling leads to reduced sporadic lateral inhibition and increased TC transmission efficacy.

**Conclusion:**

Dendritic GJs of neocortical inhibitory networks can have very powerful effects in modulating the strength and the temporal properties of sensory induced feed-forward inhibitory and excitatory responses at a very high frequency band (>200 Hz). Rapid capacitive currents are identified as main mechanisms underlying interaction between two transient synaptic conductances.

## Background

In this study, I attempt to elucidate how dendritic gap junction coupling among GABAergic fast-spiking interneurons promotes sensory processing in the primary somatosensory cortex. Although the existence of GJs was determined over 3 decades ago [1-3], the roles of GJs in sensory mediated cortical inhibitory responses are unclear. Sensory feed-forward inhibition plays important roles in shaping the responses of principal cortical neurons, constraining runaway excitation [4,5], sharpening the contour of the receptive field [6] and improving the temporal fidelity [7]. As such, TC activation of GABAergic inhibitory interneurons directly modulates the size and/or properties of the receptive field in somatosensory [8,9], auditory [10] and visual cortices [11]. In the somatosensory cortex, feed-forward inhibition is predominantly mediated by small clusters of GJ coupled fast-spiking (FS) interneurons ([12-14]. FS interneurons form GJ coupled networks in the barrel cortex [15], and other cortical regions [16,17]. Most intriguingly, dendritic GJ connections have been demonstrated to contribute to high frequency oscillations in neural networks [16,3]. In computational modeling studies, a great deal of progress has been made toward understanding the roles of GJ coupling in network synchrony [18-23]. However, no study has thus far tested how dendritic GJs contribute to sensory induced responses.

It is impossible to evaluate the contribution of GJ to sensory processing using conventional TC preparations and electrophysiological recordings because: ***1) ***high frequency thalamic stimuli often evoke aberrant cortical activities and prevent accurate study of synchrony [24,25]; ***2) ***in the barrel cortex, parvalbumin-positive interneurons located near the barrel walls and hollows receive inputs from different whiskers and thus may be asynchronous in nature [26,27]. I therefore created a computational simulation based on data from intracellularly labeled and reconstructed FS interneurons. The computational simulation approach was used in conjunction with paired recordings from brain slices in order to overcome the intrinsic shortcomings inherent in each method. The intrinsic and synaptic properties of the FS network were reconstructed based on patch-clamp recording data. The dendritic location, density and conductance of GJs were simulated based on recent anatomical studies [28,29]. The simulation of GJs, TC induced spikes and intrinsic properties were validated by patch-clamp recordings from pairs of connected FS cells in TC brain slices. In this manuscript, I examined the following questions: ***1) ***How do dendritic EPJPs interact with transient TC inputs to promote synchronization? ***2) ***How does the interaction of GJs and TC inputs contribute to the feed-forward inhibition and response properties of principal neurons? ***3) ***What are the underlying ionic mechanisms? The computational simulation and the analysis of real time electrophysiology data helps define a novel role of dendritic GJs at a very high frequency band (>200 Hz) and its underlying mechanisms. In addition, I describe a new role of dendritic GJs, i.e. dendritic GJ coupling among inhibitory networks drastically enhances sensorily induced spike transmission efficacy in excitatory neurons and promotes spike synchrony among excitatory neurons.

## Results

### Whole-cell patch clamp study I: measuring the strength of GJs from electrically coupled GAD67-GFP positive FS pairs

**Figure 1:**
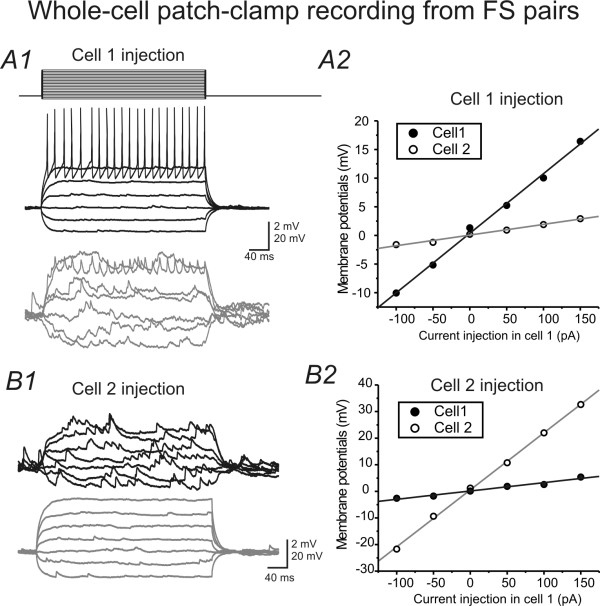
**GJ coupling in pair of FS interneurons in barrel cortex layer 4. *A1& B1)***. Current (stepwise increase) injections in cell *a *(*A1*) or cell b (*B1*) induced membrane responses of cell *a *(black) and cell *b *(gray). ***A2& B2) ***Voltage-current relationship in cells of *a *(filled circles) &*b *(open circles). Solid line: linear fits for the data sets.

To increase the recording success rate from pairs of interneurons, I used GAD67-GFP mouse in which virtually all GABAergic cells are GFP positive [30]. In the barrel cortex layer IV, the majority of recorded eGFP-positive cells exhibited high-frequency FS firing patterns (Fig 1A1). In GAD67-GFP mice, reciprocal electrical coupling was present in a majority of pairs of GFP-positive, FS interneurons (e.g. Fig. 1; 83 ± 6%, n = 12). GJ conductance has traditionally been estimated using the term 'coupling coefficient' [17]. The coupling coefficient reflects how much membrane voltage is generated in the postsynaptic cell via GJ coupling. However, the methods for estimating the coupling coefficient have not been reported in detail. Here, I describe how coupling coefficient is measured. First, I applied steps of hyperpolarizing and depolarizing currents to one of the FS cells, and recorded membrane responses in both cells (e. g. Fig. 1). As shown in Fig. 1, in GJ coupled cells, the voltage responses in the first basket cell in response to current injection were reflected in the second basket cell and vice versa. Action potentials elicited in one cell were also reflected in the other cell (Fig. 1). I next measured the membrane voltage to current relationship from the coupled FS pair (Fig. 1), i.e. the slope of V-C curve. Coupling coefficient = the slope of the membrane responses in the postsynaptic cell/the slope of the membrane responses in the presynaptic cell. Using this method, the mean coupling coefficient was 0.14 ± 0.05 (mean ± SD, n = 5 pairs). I next estimated the conductance of the EPJP, using input conductance of the postsynaptic cells multiplied by the coupling coefficient. G_GJ _= (input conductance of the postsynaptic cell* coupling coefficient). Using this method, the EPJP conductances ranged from 0.1 to 0.6 nS with a mean value of 0.4 ± 0.1 nS (mean ± SD; n = 5). In the latter part of the experiments (, the EPJP conductance value of 0.4 nS was considered as the 'physiological GJ value' and used in the modeling. As discussed later, the mean value of 0.4 nS was probably an underestimated value, due to attenuation via dendritic branches.

### Computational simulations study I: validation of GJ coupling in FS pairs

**Figure 2:**
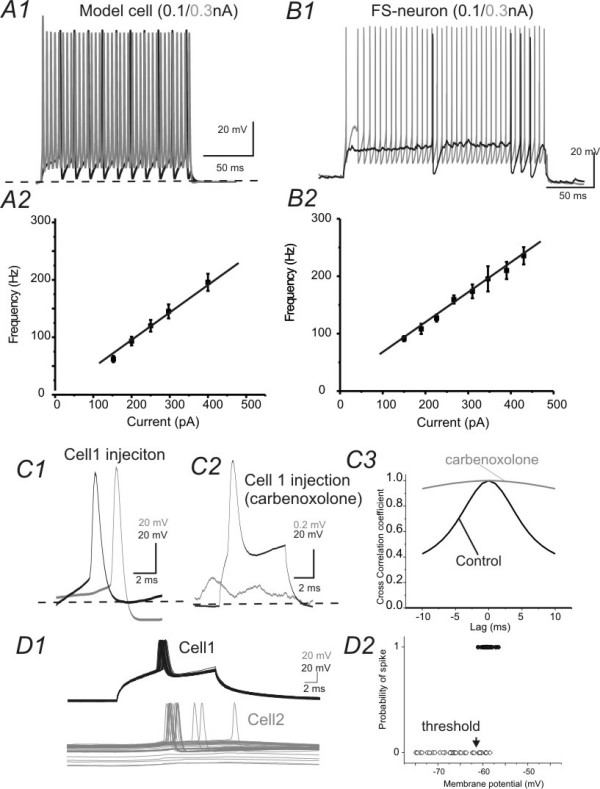
**Simulation and electrophysiological recordings of GJ-mediated responses in model and real FS pairs**. ***A1 & B1)***Repetitive spikes were evoked in a model cell (*A1*) and a real FS interneuron (*B1*). Black and gray traces were evoked by currents of 100 & 300 pA, respectively. ***A2& B2) ***F-I (frequency-current) plot of the model FS neuron **(A2) **and a real FS cell **(B2)**. ***C) ***Presynaptic (black) and postsynaptic responses in a GJ coupled FS pair recorded in slice in the absence (C1) and presence of GJ inhibitor carbenoxalone (C2). C3: Spike synchronization is strong in C1 and weak in C2. ***D) ***Representative traces (D1) and spike probability plot (D2) showing that the spike transmission depends on the membrane potential of the postsynaptic cell.

**Figure 3:**
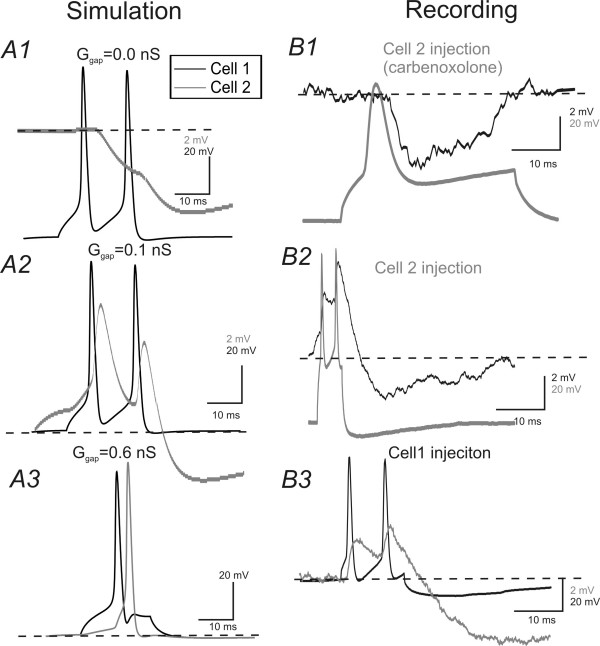
**Simulation and electrophysiological recordings of GJ-mediated responses in model and real FS pairs**. ***A1-3) ***Current injections (100 pA, 30 ms) in the model FS cell (black traces) induced postsynaptic responses (gray traces) in the postsynaptic model cell connected via GJs of varying strength (0, 0.1 and 0.3 nS). ***B1) ***AP (gray trace) in cell 2 induced IPSPs (black) in cell 1 with 50 μM carbenoxalone. ***B2 & B3) ***With carbenoxalone, current injection in cell 1 (black) or cell 2 (gray) induced initial depolarizing responses mediated by EPJPs followed by IPSPs.

The goal of the next experiment was to establish a pair of GJ coupled FS network whose properties were similar to those recorded in brain slices. I carried out the simulation using NEURON (methods, [31]. FS cells were modeled based on data obtained from whole cell recordings, where EPJP conductance was measured from membrane responses in GJ coupled pairs (e.g. Fig 1). As shown in Fig 3, in real neurons and simulated cells, the frequency and current curve for real and modeled cells was almost identical (Fig 2A2 vs. 2B2). In real patch recordings, larger GJ potentials induce synchronous spikes in postsynaptic FS neuron in 3/5 pairs that were blocked by carbenoxalone (Fig 2). The membrane potential values of postsynaptic neurons appears to be important for the spike induction. In three pairs where GJ induced spikes, a membrane potential more positive than -63 ± 3 mV is required to induce spike (Fig 2D). The GJ strength also appears to be important for spike transmission, because in the two pairs in which spike was induced (measured at holding membrane potential of -63 mV), the mean GJ values were larger (GJ = 0.47 ± 0.04 nS in spike inducing cells, n = 3 vs. 0.15 ± 0.02 nS in non-spike inducing pairs n = 2, p < 0.05). However, in other neuronal pairs, single action potential in a presynaptic FS cell only induced depolarizing spikelets (i.e. EPJPs) which were presumably mediated by GJ conductance and inhibitory postsynaptic potentials (IPSPs) that were mediated by GABA_A _receptors (Fig 3B vs. 3A). Thus these results demonstrated the validity of the simulation in spike behavior. In the model FS pair, I next altered the strength of GJ coupling and observed postsynaptic effects to presynaptic spiking (e.g. Figs. 3A1 to 3). Similar to the realistic recording obtained in the presence of carbenoxalone (i.e. Fig 3B1), when the strength of the EPJP conductance was set as 0, presynaptic spiking in a FS cell only induced hyperpolarizing GABA_A _mediated responses (Fig. 3A1 vs. 3B1)). When the strength of the GJ conductance was set to values between 0.1-0.3 nS, presynaptic spike(s) induced EPJP mediated depolarizing responses followed by GABA_A_-mediated IPSPs (Fig 3A3 vs. 3B3). The time courses of both EPJP and GABA_A _mediated responses in the model FS pair were similar to those recorded from real FS pairs (Fig 3A vs. 3B). When the strength of the EPJP was larger than 0.3 nS, a presynaptic spike usually induced a postsynaptic spike in the model cell (Fig. 3A3). These experiments ensure that the model network produced similar intrinsic and synaptic behavior as real FS pairs.

**Figure 4:**
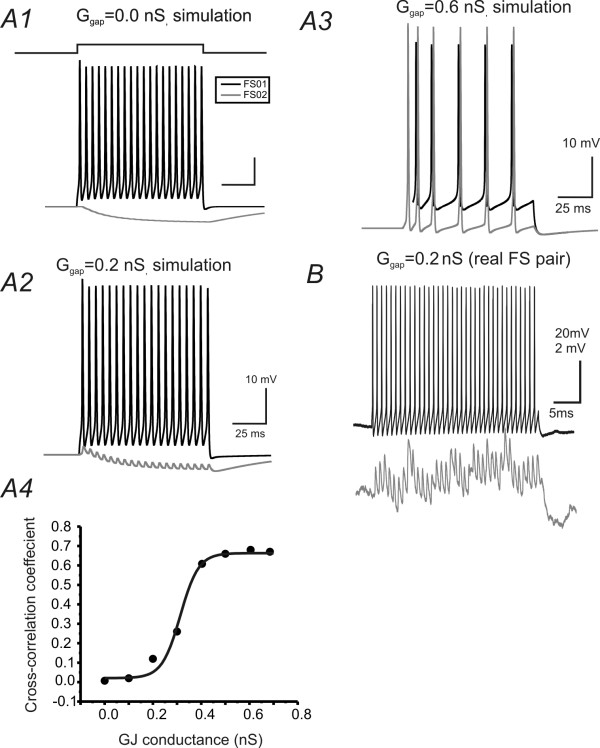
**Roles of GJs in synchronizing DC- induced spikes in simulated networks**. ***A1) ***Current (200 pA) injection in one of the GJ coupled cells (black trace) induced direct (black) and coupled (gray) responses in both cells. Cells in A1, A2 & A3 have a EPJP strength of 0 (A1), 0.2(A2) & 0.6 (A2), respectively. ***A4) ***Cross-correlation coefficient plotted against strength of EPJP conductance in the simulation (n = 10 trials). Solid line: data fitted with a Bolztmann equation. ***B) ***Recordings were made from a GJ coupled FS pair (EPJP conductance of 0.2 nS) in a brain slice.

I next evaluated the effects of increasing EPJP strength on the synchronization of spikes in the model FS pair (Fig. 4). Although these experiments have been done in many previous publications [17,32-34,15], it is necessary to compare this model network with previous works to show how EPJPs at different strength leads to spike synchronization. In the model network, current injection (200 pA, 100 ms) induced repetitive spikes in the presynaptic FS cell (e.g. Fig 4A1). At lower EPJP strength (<0.2 nS), only spikelets were induced in postsynaptic membrane (Fig 4A2). The simulated responses under this situation resembled situations recorded *in vitro *(Fig 4B vs. 4A2). When the EPJP strength was larger than 0.3 nS, postsynaptic spikes were elicited, with maximum effects occurring at an EPJP strength of 0.6 nS (e.g. Fig 4A3). I plotted the relationship between EPJP strength and the cross-correlation coefficient for spike synchronization in the model cell pairs. 50% maximum synchronization occurs when EPJP strength is 0.3 nS (Fig 4A4).

### Whole-cell patch clamp studies II: interaction of EPJPs with TC inputs

**Figure 5:**
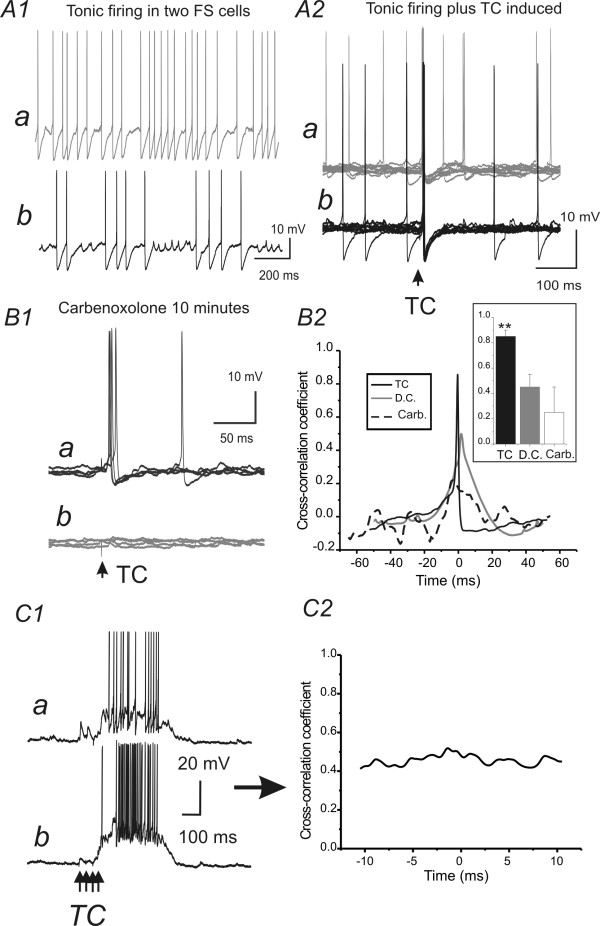
**Effects of EPJPs in synchronizing direct-current (DC) and TC-induced spikes in FS pairs of barrel cortex layer IV**. ***A) ***Tonic-spikes without (*A1*) or with TC stimulus (*A2*) in FS pairs. ***B1) ***TC- spikes in the presence of carbenoxolone (Carb.,) in the same FS pairs. ***B2) ***Cross-correlation analysis of the response of A1 (gray), A2 (black solid line) and B1 (black dotted line). Inset: comparison of the degree of synchronization induced by TC with GJ coupling (filled black bar), D.C. with GJ coupling (filled gray bar) and TC without GJ coupling (open bar), respectively. **: p < 0.01 vs. D.C. and carbenoxolone groups, respectively, n = 5. ***C1) ***High frequency TC stimulus (4 shock@25 Hz) induced responses in the same two FS cells. Note that the TC stimuli induced an "upstate" in which the membrane potentials of the two FS cells were very close to firing threshold. ***C2) ***Cross-correlation analysis of the response traces of C1.

The objective of this experiment was to exmaine properties of TC induced responses in GJ coupled FS pairs in brain slices and pharmacologically evaluate the contribution of GJ to the TC induced synchronous responses in FS pairs (if any). In mouse brain slice preparations, I first examined the synchrony of spontaneous spike trains in FS pairs. As expected, in GJ coupled pairs a modest degree of synchrony was observed (Fig 5A, cross-correlation coefficient value = 0.45 ± 0.1). The time window for the synchrony was 25 ± 5 ms (n = 10, e.g. Fig 5A2). I next examined synchrony of TC-induced spikes in FS pairs. In the same connected pairs, thalamic stimulus was applied to elicit TC-mediated spikes in FS cells. I found that the thalamic stimulus also induced synchronous firing in the connected FS pairs (Fig 5A2 &5B2). In addition, the degree of synchrony was significantly higher than the synchrony induced by direct current injection (0.83 ± 0.1 vs. 0.45 ± 0.1, n = 10, p < 0.01; Fig 5B2 inset), and the cross-correlation curve had a much shorter time window for TC induced spikes (6 ± 2 ms, n = 10, e.g. Fig 5B2). It is possible that the enhanced synchrony of TC- vs. direct current-induced spiking was due to the addition of synchronous excitatory input, because in 4 slices where 4 stimuli @50 Hz (under the same recording conditions) was applied to induce 'upstate-like' firing in FS pairs (e.g. Fig. 5C1), the synchrony was weak (Fig 5C2 vs. 5B2). In normal TC slices, high frequency TC stimulus often induces "up-state" or "epileptic" prolonged depolarization, presumably due to the truncation of axons in the slices [25]. In experimental conditions, it is impossible to dissect the contribution of EPJP and TC to network synchrony, or to determine whether there is any interaction between EPJPs and TC inputs. Nonetheless, to estimate the contribution of TC inputs alone on the spike synchrony, I applied the GJ antagonist carbenoxalone (10 μM) and studied spike synchronization. Bath application of carbenoxalone (10 μM) had a slow effect 10 minutes after it was added to the bath perfusion (not shown) and was totally reversible after 30 minutes of washout (not shown). In addition, I found no significant effects of carbenoxalone (10 μM) on the passive properties of FS cells (n = 4). As shown in Fig 5B1 &5B2, there was little synchronization in the presence of carbenoxalone. However, in all 4 pairs recorded, I could not elicit a robust TC input.

### Computational simulations study II: EPJPs, at physiological strength, can bring about reliable synchronous firing of coupled FS cells

**Figure 6:**
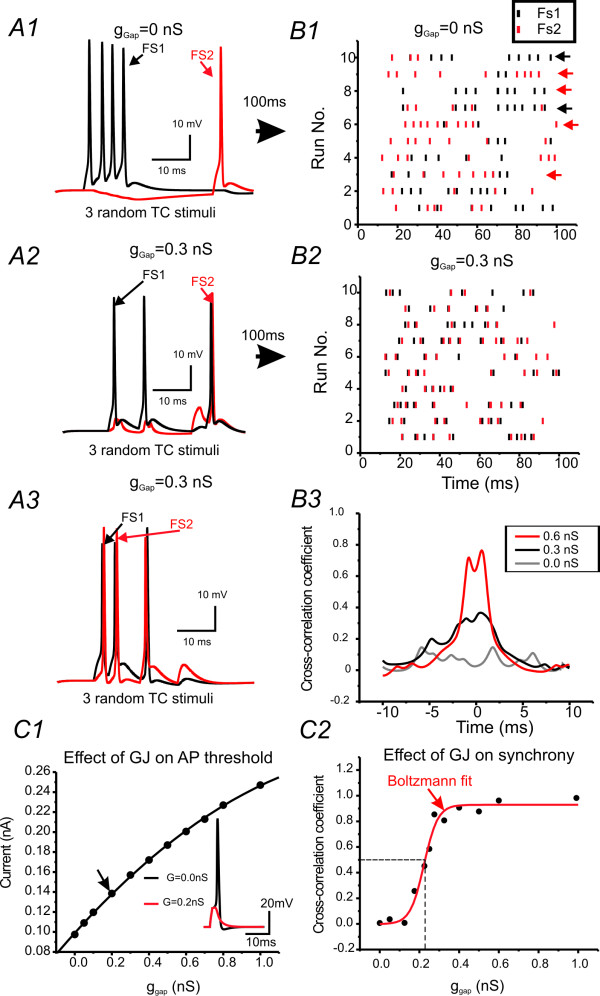
**Roles of EPJPs in synchronizing 'whisking-like' TC responses in simulated networks**. ***A) ***Membrane responses in a pair of FS cells induced by three TC inputs with variable latency to each cell. ***B1&2) ***Raster plot of a similar experiment as A1 showing the timing of spikes induced by asynchronous TC impulses in the two model FS cells without EPJP (B1) or with EPJP (0.3 nS, B2). Black and red arrows: FS cells 1 or 2 fire separately to the TC stimuli. ***B3) ***Cross-correlation analysis for evoked responses in the two model FS cells with different coupling strength (0-0.6 nS). ***C1) ***Effects of EPJP conductance on the spike initiation in model FS cells. ***C2) ***The relationship between EPJP strength and the cross-correlation coefficient was plotted. The curve is a best fit for the Boltzmann relation, CC/CC_max _= 1 + exp [(g + g_1/2_)/K]}^-1 ^where g_1/2 _= 0.22 ± 0.01 nS, R^2 ^= 0.96, Chi^2 ^= 0.007. CC: cross-correlation coefficient.

The goals of the next experiment were twofold: 1) examine effect of variable TC timing on FS synchrony 2) quantitatively examine the effects of variable GJ strength on FS synchrony. Cortical cells receive TC inputs which vary in their timing and strength. In the simulation, I created one thalamic input that extended onto the soma of both FS cells. The properties of the TC-induced spikes in the model cells were almost identical to TC-induced spikes in real FS neurons recorded in rats [13]. A single thalamic impulse induces reliable and highly synchronous spikes in the model FS pairs without any GJ connections (not shown). Next, noise in the temporal domain (see method) was added to the thalamic inputs to create variations in the timing of the inputs. Increasing the variability in the timing of the thalamic input decreased the synchronous firing in both cells and increased spike failure (e.g. Fig 6A1). Under these circumstance, the spikes of the model FS pair showed virtually no synchronization at all (Fig 6B3 gray line, n = 10 trials).

Previously, we reported that TC feed-forward inhibition was mediated by a cluster (2-3) of FS cells that were potentially coupled via GJs [13]. However, direct evidence showing how EPJPs interact with TC inputs are still lacking. In the slice preparation, repetitive thalamic stimuli elicited aberrant recurrent excitations characterized by a large barrage of asynchronous excitatory depolarization which prevented detection of TC induced single spikes [13]. These aberrant depolarizations are the result of neuronal injury and loss of inhibitory innervations in brain slice preparations. This shortcoming of the brain slice preparation prevented examination of the interaction between EPJP and TC responses. I next created repetitive, asynchronous TC impulses (i.e. 'whisking-like' stimuli, see [35,36]) in the model network. First, I applied brief 'whisking-like' stimuli composed of 3 pulses. As mentioned earlier, temporal synaptic variable was applied at the TC inputs onto FS cells. As shown in Fig. 5A1, the inputs induced responses that were entirely distinct in each of the FS cells (e.g. Fig. 6A1). There was little spike-synchronization in these trials (Fig 6B3). When the EPJP conductance was incrementally added to the simulation, the degree of spike synchrony increased accordingly (Fig 6A1-A3 &6B3). FS cells located near the center of the barrel and the septum are likely to receive inputs from different whiskers, [37,27] and therefore are likely to fire asynchronously during 'whisking-like' behavior without GJ connections. I then gradually increased the strength of the EPJPs and examined synchrony induced by the asynchronous 'whisking-like' inputs [27]. As shown in Fig 5B3, the level of synchronization increased as the strength of the EPJP increased. Whisking events can last for a couple of seconds each [27]. I next applied longer duration repetitive TC inputs (i.e. 10 Hz for 1 second). Similar to the short TC trains (Fig. 6A1-A3), a time variable was also added to desynchronize the two TC inputs. As shown in Fig 6B1(g_GJ _= 0), the spikes occurring in each FS cell were sporadic and not correlated. There was no overall correlation in the spike-timing between the two FS cells. In contrast, when a modest EPJP conductance (0.3 nS) was added to the simulation, both FS cells fired synchronously throughout the 'whisking events' in 80 ± 5% of the events (e.g. Fig 6B2, n = 10 trials). Another interesting result was the relationship between the values of g_GJ _and the spike cross-correlation coefficient, which showed a nonlinear relationship (see Fig 5C2). There was a sharp threshold conductance of around 0.2 nS, below which the dendritic EPJPs play little role in spike synchronization (Fig 6C2). This finding is similar to those of dendritic EPJPs in gamma oscillations in distributed inhibitory networks in the hippocampus [16]. These results suggest that EPJPs, at physiological strength, can bring about reliable synchronous firing of coupled FS cells and thus potentially provide reliable sensory feed-forward inhibition to the TC circuits during sensory processing. One caveat regarding the role of EPJPs in modulating TC induced responses was that EPJP increased the input conductances, therefore GJ coupled cells have a reduced excitability to weak inputs. This can be demonstrated by testing the effects of EPJP on spike initiation. As shown in Fig 6C1, the threshold current required for spike-initiation increased exponentially with the EPJP strength. In fact, because the GJs were located in the dendrites, this could have a large negative effect on excitability (see last part of the results).

### Electrophysiology and Computational simulations study III: Interaction of EPJPs with TC inputs: ionic mechanisms underlying the TC-EPJP interactions

**Figure 7:**
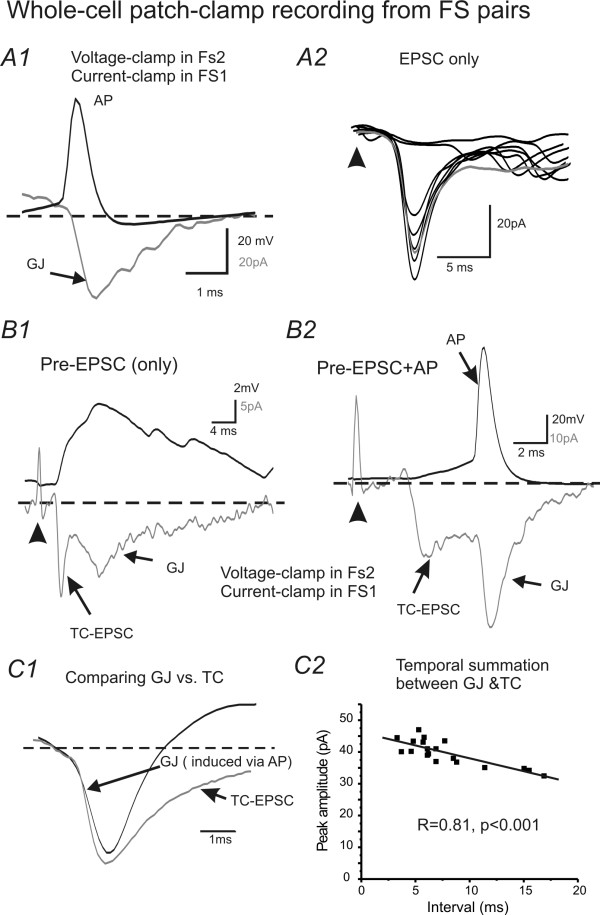
**Mechanisms underlying TC and EPJP interaction: temporal summation of EPJP and TC**. ***A1) ***Current-clamp recording of AP (black trace) and voltage-clamp recording of EPJC (gray trace) in a FS pair from brain slice. ***A2) ***Voltage-clamp recording of TC -EPSCs in a FS cell. ***B1&B2) ***A subthreshold (B1) and suprathreshold (B2) TC induced response in a presynaptic FS cell (black, current clamp) and its postsynaptic responses in another FS cell (gray, voltage-clamp), respectively. ***C1) ***Normalized conductance curve of EPJP (induced by single AP) and TC-EPSC in real FS neurons (averaged from 6 cells). ***C2) ***Temporal properties of TC-EPSC and EPJP summations measured in FS pairs. Solid line: linear fitting (R = 0.81, p < 0.001).

**Figure 8:**
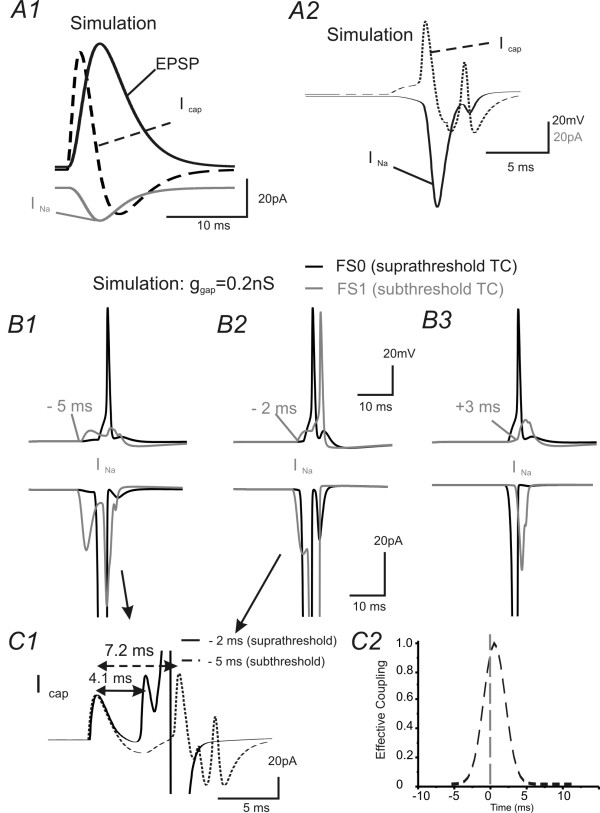
**Mechanisms of interaction of transient EPJP and EPSPs**. ***A1) ***EPSP underlying I_Cap_and I_Na _in soma of a simulated FS cell. The I_Cap _precedes the onset of I_Na_. ***A2) ***In a GJ coupled pair, a suprathreshold current induced an AP in a presynaptic FS cell (not shown), and post-junctional responses (not shown). The I_Cap _and I_Na _of the postsynaptic (via GJ coupling) cell was plotted. The two transient I_Cap _precedes the onset of I_Na _. ***B1-3) ***A critical time window for TC and EPJP interactions. Two TC inputs arriving at the GJ coupled FS pairs, respectively. A weak, subthreshold TC input arrives at FS1 (gray trace) at different time point in reference to the time of a strong, suprathreshold TC input to FS0 (black trace ***C1) ***The I_Cap _of FS1 in B2 and B1 was aligned at the onset of the two TC inputs. Note that the initial I_Cap _transients are the same (because TC input maintains the same). However, the second transient I_Cap _arriving 3 ms earlier (4.1 ms vs. 7.2 ms) and resulted in larger I_Na _(suprathreshold) in FS1 (solid line). ***C2) ***The effective coupling (number of spikes/number of TC inputs) between the two transient TC inputs was plotted as a function of interval of the two TC transients in a FS cell with EPJP of 0.2 nS.

**Figure 9:**
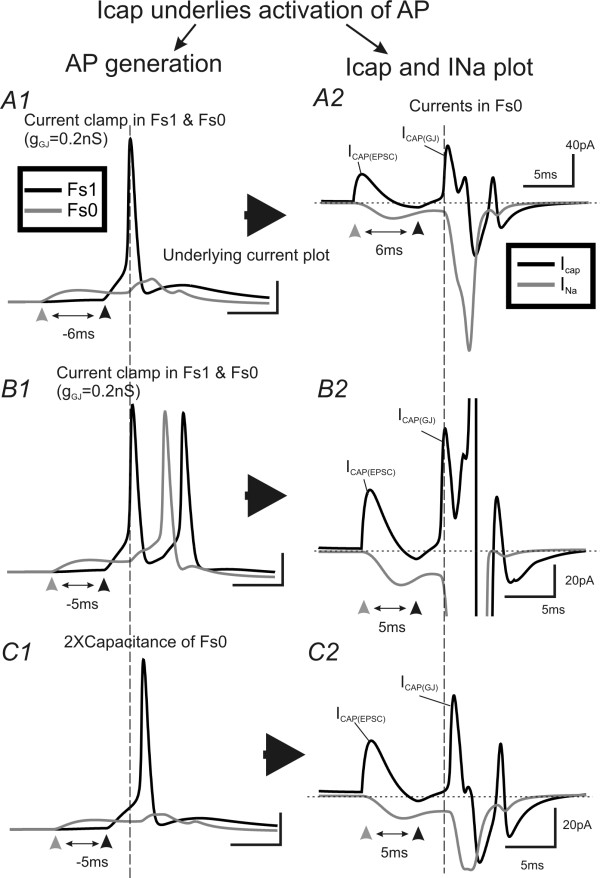
**Mechanisms of interaction of transient EPJP and EPSPs: I_cap_**. In all panels, FS0 and FS1 are coupled by GJs (g_GJ _= 0.2 nS). ***A1) ***A weak, subthreshold TC input in FS0 (gray trace) precedes (6 ms) a strong, suprathreshold TC inputs to FS1 (black trace). ***A2) ***I_Na _(gray) and I_cap _(black) in soma of FS 0. ***B1) ***The weak TC input arriving at FS0 precedes the suprathreshod TC inputs by 5 ms. Note that the same TC input now induced suprathreshold responses in FS0. ***B2) ***I_Na_(gray) and I_cap _(black) in soma of FS 0. Note that the peak of I_cap _precedes the large inward I_Na_. ***C) ***Experimental conditions in C are exactly the same with B, except that the capacitance of the FS0 was doubled. Now the same TC response failed to induce action potential in cell FS0. Note that the I_cap _in C2 was relatively smaller than B2.

In the next series of experiments, I attempt to examine how does TC and EPJP interact to enhance FS synchrony. To achieve this goal, I first performed electrophysiology experiments in real FS pairs to study temporal summation between TC and EPJP. Then I use computational simulation to examine which conductance may be important for the summation. Since spiking in neurons is induced by transient membrane depolarizations that induce rapid activation of voltage-gated sodium channels [38,39], I examined the kinetics of EPJP and TC mediated membrane conductance in real FS cell pairs (Fig. 7). Using paired recordings, I obtained EPJP and TC induced currents in the FS pair (e.g. Fig. 7A1 vs. 7A2). Time courses of the currents were then converted to conductance and normalized. The mean normalized conductance curve for EPJP and TC is shown in Fig. 7C1. As shown in Fig 7C1, the conductance curve for TC induced responses was slower than action potential induced EPJP responses. Next, I examined compound current responses (EPJP & TC) under various experimental conditions, where TC stimulus induced supra-threshold firing in only one of the FS cell pairs (the other FS cell was held at a more hyperpolarized voltage to prevent spiking, e.g. Fig. 7B). As shown in Fig 7B2, the AMPA and EPJP conductances were summated in the postsynaptic cell if they both arrived during the same brief period. I varied the interval between TC and EPJP conductance, and the temporal aspects of the EPJP and TC summation were summarized in Fig 7C2 (n = 4 FS pairs). In these experiments, the postsynaptic cells were artificially held under voltage-clamp recordings to prevent firing. In real situations, where the current can freely propagate to the axon initial segment, the interaction could potentially bring about firing in the postsynaptic cell. However, in these real cell pairs, the exact mechanisms underlying the interaction of EPJP and TC are still unknown and the exact temporal window of interaction is unclear. I next took advantage of the model network where all passive, active and synaptic conductances in the soma and dendrites can be plotted simultaneously to monitor temporal interactions. In all the variables plotted (not shown), it appeared that the transient capacitive current (I_Cap_) associated with synaptic input was involved in the activation of voltage-gated Na^+ ^channels (I_Na_) in the soma. Fig. 8B demonstrates the temporal interaction of weak subthreshold TC with EPJP to produce action potentials. In this experiment, FS_0 _received a suprathreshold TC input, and FS_1 _received a subthreshold TC input. The timing of TC input in FS1 changed incrementally in 1 ms steps from -10 ms (i.e. preceded the timing of TC in FS_0_) to +10 ms (i.e. after the timing of TC in FS_0_). As shown in Fig 8B2 &8C2, only during a very narrow time window (4 ms for 50% effective coupling, Fig 8C2), did the EPJP and TC mediated conductances summate and the subthreshold TC inputs became a suprathreshold response (Fig 8B2, n = 10 trials). Interestingly, this value (4 ms for 50% TC-EPJP coupling efficacy) was very similar to the time constant values (4.4 ± 0.3 ms, n = 16) for FS interneurons measured with patch-clamp recordings. It appears that I_Cap _plays an important role in the interaction, because it preceded the I_Na _(Fig 8C1) and the two short I_Cap _preceded the supurathreshold summation of I_Na _(Fig 8C1: 4.1 ms). To demonstrate that I_Cap _was a causal factor for inducing I_Na_, I next manipulated the value of FS capacitance while leaving all other aspects of the network unchanged. The capacitance value was doubled by increasing the surface area of the soma, which was done by modifying the somatic morphology data. A shown in Fig 9C vs. 9B, when I increased the capacitance value of the postsynaptic cells, it converted the previous suprathreshold responses to subthreshold responses, and vice versa (data not shown). By carefully examining the effects of doubling the capacitance value, I found that the I_Cap _underlying EPJP (I_Cap.GJ_) decreased by about 50% (see I_Cap.GJ _in Fig 9C2 vs. 9B2). Consequently, the I_Cap _was not sufficient to induce I_Na _sufficient for inducing action potentials (Fig 9C2). Thus I_Cap _not only precedes I_Na_, its magnitude determined the value of I_Na_, suggesting that I_Cap _is an important factor in determining action potential initiation.

### Computational simulations study IV: The effects of GJ coupling in FS network on the synchronization of spike-transmission in excitatory neuronal networks

**Figure 10:**
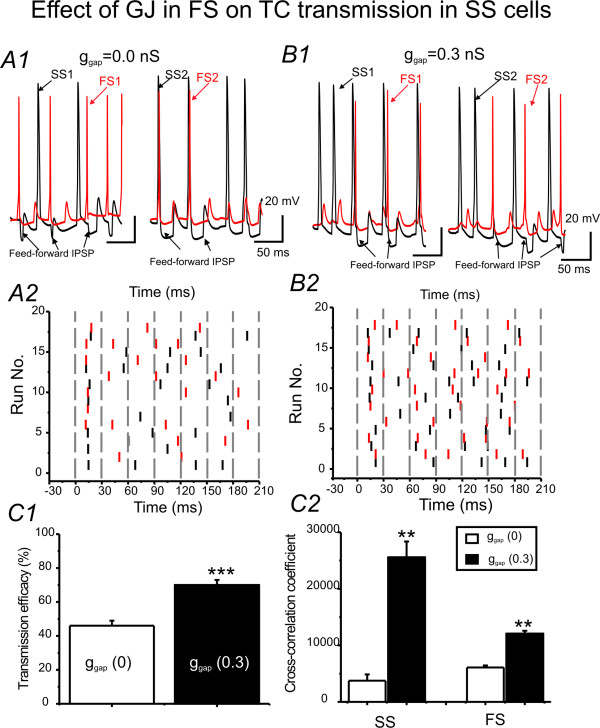
**Role of EPJP in regulating TC induced spike patterns in simulated barrel circuits**. ***A1&B1) ***TC (6 stimuli@30 ms interval) induced spikes in an simulated layer 4 network containing a FS pair (red trace) and a spiny stellate pair (SS, black trace). A1: EPJP = 0 nS between FS pairs. B1: EPJP = 0.3 nS between FS pairs. Note that feed-forward IPSP in spiny stellate neurons (indicated by black arrows) follows each successive spike in FS cells. ***A2 &B2) ***Raster plot of spikes induced by TC (6 stimuli @ 30 ms intervals) inputs in networks without GJ coupling (A3) and with modest GJ coupling (0.3 nS, B3). ***C) ***Spike transmission efficacy in SS cells (number of spikes/number of TC inputs (***C1***) and spike synchronization (***C2***) in barrel circuits with (filled bar, g_EPJP _= 0.3 nS) and without GJ coupling (open bar), ***P < 0.01; ** p < 0.01, n = 10 trials.

The above results have shown the roles of GJs in synchronizing sensory induced spiking in FS networks. What are the effects of the synchronization on spike-transmission efficacy in principal neurons? To address this question, a simulation was performed in layer IV networks containing both GJ coupled FS pairs and spiny stellate cells (method). As shown in Fig 10A1, spiking in FS cells induced postsynaptic inhibitory responses in the spiny stellate cells (feed-forward IPSPs). I compared the spike-transmission efficacy in networks with varying EPJP conductance in FS interneurons. The spike-transmission efficacy was calculated as the number of spikes in spiny stellate cells/the number of TC inputs. As shown in Fig 10C1, the transmission efficacy increased significantly at an EPJP value of 0.3 nS (35 ± 5% enhancement, p < 0.01, N = 15 trials). Note that the number of TC- induced spikes in the two spiny stellate cells increased significantly when FS cells in the network were coupled by GJs (e.g. Fig. 10B2 vs. 10A2, and 10C). In addition, the spike synchrony in spiny stellate cells increased as a function of synchrony in FS cells (Fig 10C, 490 ± 20% increase in spiny stellate cells vs. 105 ± 8% increase in FS pairs, p < 0.01, n = 10), suggesting that GJs promote signal transmission efficacy and synchrony in excitatory networks (spiny stellate cells) by reducing sporadic and un-correlated spikes in FS cells (e.g. Fig 10B2 vs. 10A2). These results suggest functional importance for GJs in regulating receptive field properties.

**Figure 11:**
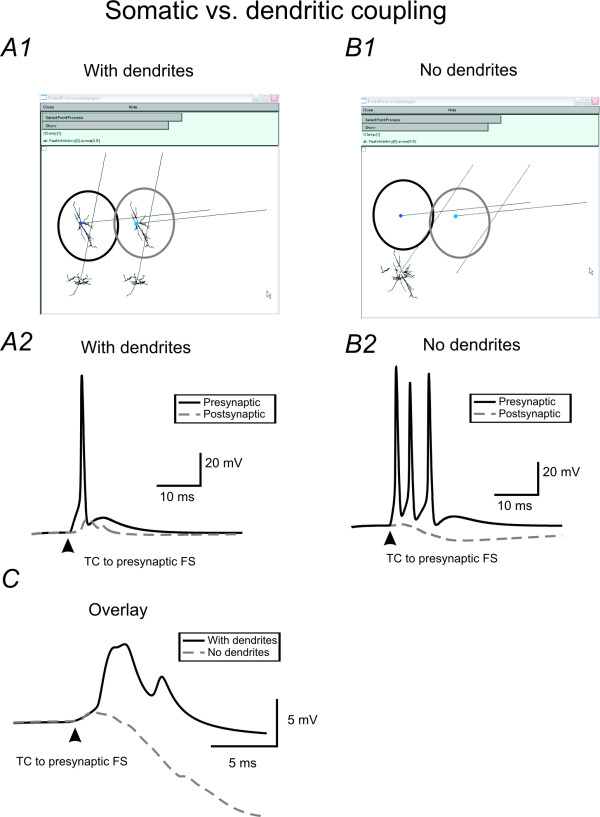
**The value of GJ mediated conductance in real neuron may be underestimated due to dendritic coupling**. ***A) ***The single cell responses in FS1 &FS0, induced by a TC stimulus applied to FS1. FS1 &FS0 were coupled via GJ (g_GJ _= 0.4 nS). ***B) ***Experimental conditions in B are exactly the same as A except that the dendrites were removed (see B1). Note that without dendrites (and dendritic GJs), the same TC stimulus induced responses in FS1 are more excitable, however, in the postsynaptic FS cell, the GJ coupled responses are smaller. ***C) ***Overlay of the GJ coupled responses in the postsynaptic FS cell, showing that the dendritic responses are ~7 fold larger with dendrites.

As described earlier, the values of GJ coupling in real neurons was measured by paired recording techniques from the cell soma. Although anatomical studies have identified a dendritic distribution of GJs, due to technical reasons, it is difficult to make dual recordings from dendrites of coupled FS cells. Due to attenuation along dendrites, GJ conductance values may be underestimated using somatic measurements. In simulation experiments, dendritic branches can be easily removed (Fig 11A1 vs. 11B1). I thus examined the effects of removing or adding dendrites on somatic EPJP potentials. EPJPs in the soma area of a FS cell with or without dendritic trees were measured and compared. As shown in Fig 11, adding dendrites to the simulation increased the EPJP value 3.5 ± 0.4 fold (see Fig 11C). In addition, adding dendrites decreased the excitability as previous described (see Fig 6C1). In this study, although dendritic GJs have been included in the simulation, the value of GJ conductances was based on measurements from paired recordings from the FS soma. Thus dendritic GJs may have larger effects on spike synchronization and modulation of receptive field properties than described by this study.

## Discussion

### FS cells and sensory feed-forward inhibition

In visual and auditory cortices, feed-forward inhibition serves to increase the temporal and spatial precision and thereby reduce the randomness of cortical operation [10,40,11]. Although the neuronal groups involved in the feed-forward inhibition are not entirely clear, it has been suggested that FS basket cells are involved [41,12-14]. In the somatosensory cortex, both excitatory and inhibitory neurons in barrel cortex are activated by TC inputs [42,9,44,41]. There is both synchronous activation of populations of glutamatergic neurons by TC afferents and abundant coupling among these excitatory neurons. This could potentially lead to aberrant recurrent excitation and prevent precise detection of subsequent sensory inputs [45,25]. Strong and reliable feed-forward inhibition onto excitatory neurons in layer IV must be present to effectively "shunt" recurrent excitation and preserve discrete signaling in cortical networks. In barrel cortex, di-synaptic feed-forward inhibition, mediated by direct TC excitation of interneurons, is a critical part of the sensory gating process [41,8,12,9]. Studies obtained from barrel cortex *in vivo *suggest that subgroups of interneurons, presumably FS interneurons, known as suspected interneurons (SINs), are a major candidate for providing the feed-forward inhibition [12]. In an earlier study, we have shown that a small cluster of FS neurons (2-3) mediates TC feed-forward inhibition in each spiny stellate neuron and can powerfully shunt TC-mediated excitation [13,53]. The activation of FS cells by TC inputs, coupled with powerful feed-forward inhibition from these neurons, would profoundly influence sensory processing and constrain runaway excitation *in vivo *[13,35,36,14]. In addition, the activation of coupled FS cells by strong TC inputs is likely to play a very important role in sharpening the contour of the receptive field [6] and improving temporal fidelity [7]. In summary, these earlier studies have established a major role of FS cells in mediating sensory feed-forward (or lateral) inhibition in somatosensory cortices.

Enormous progress has been made toward understanding the roles of GJs in both experimental studies [46,47] and in simulation studies [18-23,48]. Functionally, the most unique attribute of electrical synapses is their bidirectionality. Electrical synapses often interconnect neurons of similar type, size, and input resistance, primarily using Cx36-dependent GJs [46,47]. In neocortical inhibitory networks, small signals that are slow, such as after-hyperpolarizations, burst envelopes, or subthreshold oscillations can all effectively propagate via GJs [48] and enhance the synchrony of interneuronal networks. The ability of GJs to synchronize pairs and populations of neurons has been studied extensively by a number of groups, particularly using modeling and analytical approaches [18-23,48]. In some of these studies, GJs have strong effects on network behavior in frequency ranges from 10 Hz to 50 Hz [48]. In other studies [49], GJs exert an effect over large frequency ranges (10-130 Hz). So far, it is unclear whether EPJPs interact with TC, and if so how they interact, the present study is the first of its kind to address these important questions.

### EPJPs TC interaction occurs in an unprecedented high frequency band

GJs have generally ***not ***been considered as playing a role in synchronizing TC mediated inhibitory responses within the millisecond range (>200 Hz). The reason why this might be important is that many FS interneurons are located near the barrel borders, these cells may be driven by convergent TC inputs derived from different thalamic barreloids [27,50], in which sensory inputs have very small temporal differences (< a few milliseconds, cf. [51]). Another reason is that the repetitive excitatory inputs in interneurons exhibit very robust filtering properties that are due to short-term presynaptic plasticity and postsynaptic summations [52,24]. It is unclear whether GJs play any role in this process. In an earlier study, we have shown that TC-mediated feed-forward inhibition is mediated by small clusters of FS cells [13]. However, under the recording conditions described in that study, highly synchronous and supra-threshold TC inputs were provided to induce feed-forward inhibition in those cells. My results show that dendritic EPJPs, at physiologically relevant strength, play a critical role in synchronizing TC-mediated spikes in FS cells (Fig 6B). I also carried out experiments to further delineate mechanisms underlying EPJP and TC summations. Specifically, experiments shown in Figs 5, 7, 8 &9 helped to address these questions: To what extent is the synchrony in suspected interneurons (as seen in *in vivo *experiments, e.g. [12]) due to TC input and to what extent is it due to GJ coupling? How do TC inputs and EPJP coupling interact? In pharmacological experiments using the GJ antagonist carbenoxolone, my results showed that synchrony induced by EPJP and TC alone contribute to 50% and 25% of the overall synchrony in brain slices (Fig 5B2), respectively. However, the percentage numbers may be misleading because these conductances need to interact to produce the enhanced synchrony and no synchrony was produced when they didn't interact (e.g. Fig 5C vs. 5B or 5A). Also taking advantage of the model network where all passive, active and synaptic conductances in the soma and dendrites can be plotted simultaneously to monitor temporal interactions, I have been able to identify a critical time window in which transient capacitive current (I_Cap_) associated with synaptic input is involved in the activation of voltage-gated Na^+ ^channels (I_Na_) in the soma (Fig 8 &9). I have shown that in GJ coupled FS pairs, temporal summation depends on the amplitude and the interval of the I_Cap_. Another significant finding was the importance of dendritic GJ coupling. The role of dendrites in GJ coupling has been examined in detail in several earlier studies [53,22,23]. All these studies emphasized the importance of distal dendritic coupling, my results certainly support these ideas. Saraga et al, described a phase delay effect of dendritic coupling, which is a very interesting phenomenon and deserves careful further study. One caveat of current study is related to the location of GJ in the specific compartment of dendrites. So fat, there is no experimental data regarding recordings of GJ from different dendritic locations of interneurons, there is also no anatomical evidence to show that GJ is restricted to certain dendritic compartment. Therefore, the current model did incorporate this scenario. However, based on results presented in Fig 11, one can predict that if this is indeed the case (i.e. GJ highly restricted to proximal dendrites), the dendritic coupling is not as effective as those in the distal dendrites, a result between those shown in Fig 11A2 &11B2.

### Implications for understanding the role of GJs in modulating the receptive field properties

Another significant finding is that I provide an estimate of the effects of enhanced coupling on firing in spiny stellate cells, which to the best of my knowledge, has not been done in simulation or electrophysiological studies. By studying the temporal domain of the EPJP and TC interaction (Figs 7 &8), the experiments suggest that ***1) ***stronger TC-mediated responses in one of the FS cells play a key role in synchronizing the spiking in GJ coupled FS networks, ***2) ***the firing pattern of a FS cell which receives relatively weak TC inputs should be dominated by other GJ coupled FS cells that receive robust TC inputs (e.g. Fig 8B2). Based on these hypotheses, it is also conceivable that when both GJ coupled cells receive modest, near threshold TC inputs (i.e. sub-threshold inputs), these inputs can induce firing only if they produce temporal summation via EPJP overlap (within ~4 ms from peak of TC or EPJP) to reach firing threshold [54]. These data also suggest that the spiking behavior of the interneurons, which receive weak inputs from multiple whiskers with different receptive fields, will be determined by other GJ coupled FS interneurons that receive strong TC inputs (principal whisker or receptive field, e.g. Fig 8B2 &7C1). However, this conclusion should be further validated with multi-electrode recordings *in vivo*. GABA_A _mediated responses are also generally thought to play a very important role in network synchrony in simulation [55] and in slices [56]. In the simulation and in my recordings, GABA_A _mediated IPSCs were incorporated (see methods & Fig 10). IPSCs made additional contributions to the novel spike synchronization discussed here. Specifically, IPSCs can be coupled via dendritic GJs to affect network behavior [18,57]. Because recurrent inhibitory inputs between interneurons were relatively small, compared with feed-forward inhibitory inputs onto spiny stellate neurons [58,13], the GABA_A _mediated inhibitory responses are thus likely to play a larger role in regulating spike fidelity in spiny stellate cells which has been shown in Fig 9. Indeed, in small networks containing both FS and spiny stellate cells, synchrony in FS pairs by GJs can improve TC- spiny stellate spike transmission efficacy significantly (p < 0.01) by about 35% (Fig 10C) and improve spike synchrony in spiny stellate cells by ~4 fold (Fig 10). This striking enhancement of spiking probability in spiny stellate cells by GJ in FS networks is an important novel component of the cortical mechanisms underlying sensory processing.

## Conclusion

1. Under physiological conditions, EPJPs interact with TC inputs within an unprecedented few milliseconds (i.e. over 200 Hz) to enhance the firing probability and synchrony of coupled FS cells.

2. Dendritic GJ coupling allows a drastic four fold increase in synchrony and a significant enhancement in spike transmission efficacy in excitatory spiny stellate cells.

3. The model revealed the following novel mechanisms: ***1) ***rapid capacitive currents (I_cap_), which were induced by EPSP and EPJP, underlying the activation of voltage-gated sodium channels; ***2) ***there was less than 2 milliseconds (± 2 ms for 50% coupling efficacy) in which the I_cap _underlying TC input and EPJP was coupled effectively; ***3) ***there was a threshold value at around 0.2 nS, below which EPJP had little effect on spike synchrony in coupled FS pairs; ***4) ***cells with dendritic GJs had larger input conductance and smaller membrane response to weaker inputs; ***5) ***synchrony in inhibitory networks reduced sporadic lateral inhibition and increased TC transmission efficacy. I conclude that the dendritic GJs of neocortical inhibitory networks can have very powerful effects in modulating the strength and the temporal properties of sensory induced inhibitory and excitatory responses at a very high frequency band (>200 Hz). Rapid capacitive currents are identified as main mechanisms underlying interaction between two transient synaptic conductances.

## Methods

### Animals and treatment groups

Transgenic glutamate decarboxylase (GAD) 67-green fluorescent (GFP) (Δneo) mice in which GFP is selectively expressed under the control of the endogenous GAD67 gene promoter [30] were used for electrophysiology recordings. In this strain, virtually all (~95%) GABAergic neurons expressed GFP (Fig. 1 cf. [30]). In barrel cortex layer 4, 82% of the eGFP-positive neurons are FS and parvalbumin-positive, basket cells (Fig. 1, cf. [59]), and the rest are predominantly regular spiking non-pyramidal (RSNP) cells.

### Brain slice preparations and electrophysiological recordings

GAD67-GFP mice were deeply anesthetized and decapitated. The brains were quickly removed and placed into cold (~4°C) oxygenated slicing medium containing (in mM): 2.5 KCl, 1.25 NaH_2_PO_4_, 10.0 MgCl_2_, 0.5 CaCl_2_, 26.0 NaHCO_3_, 11.0 glucose, and 234.0 sucrose. TC slices were prepared according to methods described by Agmon and Connors [60]. Tissue slices (300-400 μm) were cut using a vibratome (TPI, St. Louis, MO), transferred to a holding chamber, and incubated (35°C) for at least 1 hour. Individual slices were then transferred to a recording chamber, fixed to a modified microscope stage, and allowed to equilibrate for at least 30 min before recording. Slices were minimally submerged and continuously superfused with oxygenated physiological saline at the rate of 4.0 ml/min. The physiological perfusion solution contained (in mM): 126.0 NaCl, 2.5 KCl, 1.25 NaH_2_PO_4_, 1.0 MgCl_2_, 2.0 CaCl_2_, 26.0 NaHCO_3_, and 10.0 glucose. Solutions were gassed with 95% O_2_/5% CO_2 _to a final pH of 7.4 at a temperature of 35 ± 1°C. The method for identification of the barrel subfield in living TC slices was described in earlier studies [13,59]. A low-power objective (2.5×) was used to identify barrels and thalamic nuclei, and a high-power water immersion objective (40×) with Nomarski optics and infrared video was used to visualize individual neurons. Recording pipettes were pulled from capillary glass obtained from World Precision Instruments (M1B150F-4), using a Sutter Instrument P80 puller, and had tip resistances of 2-5 MΩ when filled with the intracellular solutions below. A Multiclamp 700B amplifier (Axon Instruments, Foster City, CA) was used for voltage-clamp and current clamp recordings. Patch pipette saline was modified according to Brecht and Sakmann [42] and composed of (in mM): 100 K-gluconate, 10.0 phosphocreatine-Tris, 3.0 MgCl_2_, 0.07 CaCl_2_, 4 EGTA, 10.0 HEPES, 4.0 Na_2_-ATP, and 1.0 Na-GTP, pH adjusted to 7.4 and osmolarity adjusted to 280 mosMl^-1^. Neurobiotin (0.5%; Vector Labs) was regularly added to the patch pipette solution. Current and voltage clamp protocols were generated using PCLAMP9.2 software (Axon Instruments).

### Simulation using NEURON

**Figure 12:**
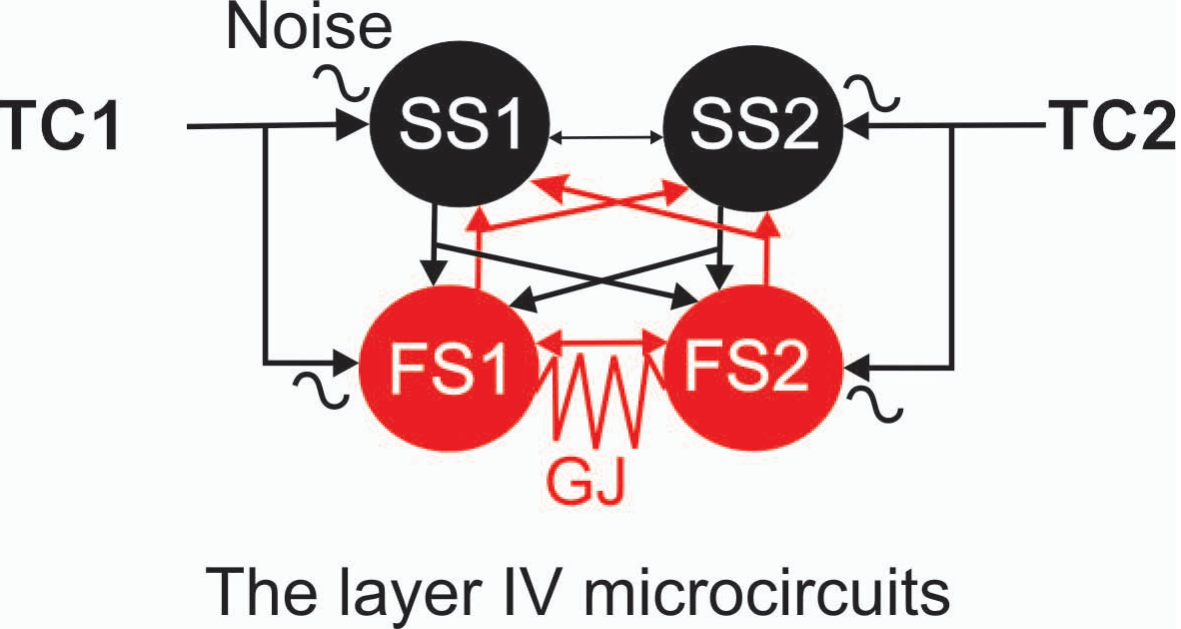
**Construction of the layer IV microcircuits in simulation**. Schematic graph shows how layer IV microcircuits were constructed in the simulation for experiments shown in Fig 10. Noise (jitter of strength and timing) were added to TC inputs. FS cell pairs were connected via both GABAergic chemical synapse (gray arrows) and GJ. SS cell pairs were connected via glutamatergic synapses (black arrows). FS-SS cells were connected via glutamatergic synapses (black arrows) and GABAergic chemical synapse (gray arrows).

All the simulations were carried out with the NEURON simulation program, version 5.9 [31]. The canonical model in all simulations consists of two identical FS cells connected via GABA_A _synapses and two identical spiny stellate cells connected via glutamatergic synapses, and two separate sets of TC inputs onto the FS cells and SS via AMPA and NMDA mediated synapses (Fig 12). Both FS and spiny stellate cells consist of an active somatic compartment and passive dendritic compartments. Each cell was implemented with an axon, soma, and a number of dendrites. The geometry of soma and dendrites of FS and SS cells were constructed using neurolucida data and was based on realistic neurobiotin labeled basket cells and spiny stellate cells. The 3D morphology data (.asc files) of dendrites was then imported to NEURON through NEURON's Import3D tool. The dendritic compartments were made from 37 dendritic segments in the FS cell. For their electrophysiological properties, uniform passive properties were used, with Ra, τm, and Rm and Cm adjusted to realistic values for FS and spiny stellate cells (details see below). Incorporating the biophysical mechanisms in the model was provided by NMODL [31]. For example, the leak current density was given by ileak = gleak (Vm - Eleak), where Vm was membrane potential. In the soma area, in addition to passive (i.e. leak) conductance, the following conductances were included in the model: a voltage gated calcium conductance (gcabar); a voltage-gated Na^+ ^conductance (gnabar), a calcium-activated potassium conductance (gkcbar) and a transient potassium conductance (IA, gabar). The calcium dependent potassium conductance was determined by intracellular calcium concentration which was dynamically regulated by voltage-gated calcium conductance and was determined by the NMODL [31]. All above conductances were distributed over the soma surface of both cell types (spiny stellate & FS). The conductances of FS cells were specificed as follows: gcabar_spike = 0.0015 nS/cm^2^, gkbar_spike = 0.018 nS/cm^2^, gabar_spike = 0.054 nS/cm^2^, gkcbar_spike = 0.000065 nS/cm^2^, gnabar_spike = 0.10 nS/cm^2^. In the dendritic compartments, only leak conductance was present, with g_leak _= 0.00045 nS/cm^2^. It should be noted that several additional mechanisms were not included in my model, for example, persistent Na^+ ^conductance, other voltage-gated or inwardly rectifying K^+ ^conductances [19], voltage-gated Ca^2+ ^conductance. The reason why I did not include these conductances was that these conductances may modulate spike synchrony. To effectively evaluate the role of GJs on action potential coupling, I only included conductances which were necessary for generation of cell-specific spikes. It would be interesting to include those additional cell properties in a future study. Membrane biophysics of the FS cells is based on data reported by Beierlein et. al., 2003 and Sun et al., 2006. For each cell type (FS and spiny stellate) the resting potential, input resistance, membrane time constant, action potential half-width, amplitude of afterhyperpolarizations and firing-rate at threshold were closely matched to those published by Beierlein et al., 2003 and patch clamp recordings from mouse FS cells (Figs 1, 2, 3,). Briefly, the biophysical properties of the FS cells are: resting membrane potential, -66 mV or -50 mV; input resistance, 210 MΩ; membrane time constant, 6 ms; action potential half width, 0.5 ms; steady-state firing rate at threshold, 55 Hz; maximal steady-state firing rate, 150 Hz; spike frequency adaptation index: 1.1. Synaptic connection characteristics were based on data provided by Sun, Huguenard and Prince, 2006 [13] and [61,56]. Briefly, TC excitatory postsynaptic potentials (EPSCs) in FS cell: amplitude (mV) 2.2-5.0 mV, rise time: 0.4 ms; decay time: 1.5 ms, coefficient of variance (CV): 0.2. Recurrent GABA_A _mediated inhibition in FS cell: amplitude: 1.5-2.5 mV, rise time: 2.0 ms; decay time: 37 ms, CV: 0.4.

### Implementation of synaptic connections in the simulation

In the simulation, thalamic input was a spike generator. On zero-crossing a signal was sent to the designated synapse at a designated weight and delay. All the connection functions (e. g. TC to spiny stellate cells) utilize the rconnect() function--proc rconnect(){//Usage: rconnect (source-cell, target-cell, target-section, rtype,//weight, delay, threshold)//rtype = 0 = AMPA ||| 1 = NMDA ||| 2 = GABAa}. The number and interval of thalamic inputs were controlled by user from a thalamic input panel "NetStim [0] at thalamic [0].soma(0.5)". To vary the thalamic spike timing, a 'noise' function was also provided on the thalamic input panel, where fractional noise, 0 < = noise < = 1, means that an interval between spikes consists of a fixed interval of duration (1 - noise)*interval plus a negexp interval of mean duration noise*interval.

### Implementation of GJ coupling in the simulation

GJ modeling methods were adopted from Migliore et al., 2005. Briefly, I modeled the current generated by GJs as I_GJ _= g_GJ_·(v_post _- v_pre_), where g_GJ_, v_post_, and v_pre_, were the GJ conductance, and the post- and pre-synaptic membrane potential, respectively. The dendritic location of GJs and the total g_gap _conductance were based on published results obtained in FS interneurons in the somatosensory cortex [33,29] and visual cortex [28]. GJs were created between the homologous anatomical structures (segments) of the two FS cells. The FS dendrites consist of 37 segments dend [0]-dend[36]. The soma is a single segment. So there are a total of 38 gap junctions. This code connects the dendrites: for i = 0,36{ gap [i] = new Gap(); FS [0].dend [i] gap [i].src(0.5); FS[1]. dend [i] gap [i].target(0.5)}; So FS [0].dend [0] is connected to FS[1]. dend [0] ... As the code was written the function set_gapg() sets the conductance to the same value for all gap junctions. Cross Correlation Analysis Tool was embedded in the NEURON model program and implemented as a simulator tool which helps analyze data from simulations. In all experiments, cross-correlation analysis were performed on two traces, with time window of analysis varied between different experiments, ranging from 10 ms to 1 sec. Complete correlation have a cross-correlation coefficient of 1.0.

### Statistics

Paired (for comparison between pre and post-stimulation in the same animals; or two treatments) and unpaired Student's T-test were used to examine statistical significance between groups.

## Authors' contributions

QQS designed and carried out the experiments and wrote the manuscript.
